# Efficacy of fluralaner flavored chews (Bravecto®) administered to dogs against the adult cat flea, *Ctenocephalides felis felis* and egg production

**DOI:** 10.1186/s13071-015-0965-4

**Published:** 2015-07-11

**Authors:** Michael W Dryden, Vicki Smith, Tashina Bennett, Lisa Math, James Kallman, Kathleen Heaney, Fangshi Sun

**Affiliations:** Dept. of Diagnostic Medicine/Pathobiology, Kansas State University, Manhattan, KS 66506 USA; Merck Animal Health, 2 Giralda Farms, Madison, NJ 07940 USA

**Keywords:** Flea, *Ctenocephalides felis felis*, Cat flea, Dogs, Fluralaner, Egg production, Adulticide activity, Control

## Abstract

**Background:**

Fluralaner is a potent insecticide and acaricide with rapid and persistent efficacy. This study measured the efficacy of fluralaner flavored chews (Bravecto®, Merck Animal Health) administered to dogs against adult *Ctenocephalides felis felis* and egg production.

**Methods:**

Twelve purpose-bred dogs were randomly allocated to two groups of six dogs each. Dogs in treatment group 1 were administered a single fluralaner flavored chew to achieve a minimum dose of at least 25 mg/kg while treatment group 2 served as untreated controls. On Days −2, 28, 56, 84, 91, 98, 105, 112, and 120 post-treatment, each dog was infested with approximately 200 unfed cat fleas, *C. felis felis* (KS1 strain). Forty-eight hours after treatment and 48 h after each infestation, eggs were collected over a 3-h period, counted and viability determined. Dogs were combed to remove any remaining fleas.

**Results:**

Treatment of dogs with oral fluralaner provided a 100 % reduction in flea counts 48 h after treatment and within 48 h of every post-treatment infestation through Day122. Egg production from fluralaner treated dogs was reduced by 99.9 % (two eggs from one dog) within 48 h after treatment and not a single egg (100 % efficacy) was thereafter collected from treated dogs. Adult flea counts and egg production from the fluralaner-treated dogs were significantly lower than for non-treated controls at all post-treatment evaluations (P < 0.001). The two eggs collected from the single treated dog 48 h after treatment did not produce any adult fleas. As no additional eggs were collected from treated dogs, no viability assessment was performed.

**Conclusions:**

A single oral dose of fluralaner flavored chews provided 100 % efficacy against repeated flea infestations on dogs for 4 months. Fluralaner reduced egg production of activity reproducing female fleas by 99.9 % and then killed every single female flea before any eggs could be produced following each subsequent re-infestation for the entire 122-day evaluation period.

## Background

The cat flea, *Ctenocephalides felis felis*, is well adapted for infesting in-home environments due to its prolific reproduction. A home can become seeded with overwhelming numbers of life stages over the course of a few weeks. Within 24–48 h of initiating feeding on a mammalian host, mating occurs and egg production begins with each female flea rapidly filling the environment with hundreds to potentially thousands of eggs during a lifespan that can exceed 100 days [1, 2]. These eggs then cycle through larvae and pupae stages before emerging as adults to begin the process anew.

Flea control management was revolutionized with the introduction of modern topical residual flea adulticide products such as imidacloprid and fipronil in the mid-1990s. Since then numerous additional products have been developed for use in various topical and, more recently, oral formulations [3, 4]. These products succeed in providing effective flea control in one of two ways; as adulticides that kill the female flea before she has an opportunity to lay eggs, and/or by negatively impacting the viability of the eggs produced [5–7]. Successful flea control requires a residual adulticide product that persistently kills newly arriving adult fleas before egg laying begins. Success can only be achieved if the residual adulticide product works for the full duration of its intended use with 100 % efficacy, because any viable female fleas surviving beyond 24 h can perpetuate the infestation [5, 6]. If limited egg production occurs in spite of treatment, then it may still be possible to control flea reproduction if the product also prevents egg development [5, 7, 8]. Under the ideal environmental conditions often found in homes, a flea typically cycles through its life stages over the course of 3 – 8 weeks, although this can occasionally extend to 90 days or rarely longer [5, 6, 9, 10]. Thus, to eradicate an infestation successfully, effective control must be maintained for at least 12 weeks. This duration will lead to clean up of juvenile flea life stages in the environment, and usually requires successful diligent administration of 2–3 or more doses of a monthly flea control product [5, 6, 9, 10].

Even with the availability of modern flea control products, fleas continue to infest the homes of dog owners, possibly because the owners do not correctly adhere to treatment recommendations [11] or because the residual speed of kill of the product in use tapers off at the end of the month permitting successful flea reproduction [12] and continued infestation. It has been reported that on average dog owners only administer 5.2 months of flea and tick preventive per year [13]. This lack of compliance and adherence puts dogs and owners at continuous risk of flea infested homes and places dogs at greater risk of exposure to vector borne diseases.

Fluralaner is a novel drug in the isoxazoline class recently introduced in an oral formulation to companion animal medicine for flea and tick protection in dogs [14, 15]. It is rapidly absorbed and widely distributed with a long half life of 12 – 15 days [16]. It appears there is minimal if any metabolism as it is primarily excreted unchanged in the feces [16]. Fluralaner’s safety profile has been thoroughly evaluated, with no adverse events noted in 8 week old puppies administered up to 5 times (280 mg/kg) the maximum deliverable dose within the dogs weight bracket (56 mg/kg) every 8 weeks until beyond the time when a steady state is achieved (2–3 administered doses) [17]. No adverse events were noted in MDR 1 homozygous negative collies administered three times the recommended dose [18]. Because of its safety profile, fluralaner can be dosed at 25–56 mg/kg which achieves a 12 week label duration of activity against fleas and multiple species of ticks (for example, in the US, *Ixodes scapularis*, the black-legged tick, *Dermacentor variabilis*, the American dog tick, and *Rhipicephalus sanguineus*, the brown dog tick and 8 weeks against *Amblyomma americanum*, the lone star tick [15, 19]).

The objective of this study was to evaluate the residual efficacy of a single dose of fluralaner (Bravecto®, Merck Animal Health) flavored chews administered to dogs against adult *Ctenocephalides felis felis*, egg production and the development of eggs to viable adults for 4 months.

## Methods

### Animals and housing

Sixteen purpose bred dogs (8 male:8 female) were studied. Physical examinations were conducted by a licensed veterinarian prior to the initial flea infestation and dogs were determined to be in good health and free of any pre-existing dermal lesions. No drugs, baths, shampoos or pesticides were administered to the dogs during the 6-day pre-conditioning phase or the course of the study other than what is described in the protocol. Dogs were fed a commercial dry ration, water was available *ad libitum* and dogs were housed in individual kennels. All animal care procedures conformed to guidelines established by the Institutional Animal Care and Use Committee at Kansas State University (IACUC # 3463).

### Animal selection and randomization

On Day −5, all dogs were infested with 100 adult cat fleas, *C. felis felis*, (KS1 strain) 1 to 5 days post emergence. On Day −4, flea comb counts were performed to evaluate the susceptibility of each dog to maintain experimental infestations and for random allocation of the dogs to the treatment groups. Each dog was combed with a fine-toothed flea comb having 12 to 13 teeth/cm. Flea removal was achieved by combing each dog thoroughly for 10 min. If five or more fleas were recovered during this period, the dog was combed for an additional 5 min. If any fleas were recovered during the second combing period, the dog was combed for an additional 5 min, for a maximum of 20 min. The six females and six males with the highest flea counts were retained for the study.

Within each gender the six dogs were ranked in descending order by flea count. For each of the six dogs in each gender group a random number generated by EXCEL was assigned to each dog in rank order. Dogs were grouped into replicates of two based on descending flea counts. The two dogs within each of the replicates were allocated to treatment groups (1 and 2). Each replicate contained one dog in each of the two treatment groups. The highest random number in each block was assigned to treatment group 1 and the other to treatment group 2. This was repeated for each block of dogs in the study. Thus, at the end of the process, there were six dogs (three male:three female) in each treatment group.

### Infestations and treatment

Dogs in treatment group 1 were administered a single fluralaner flavored chew (BRAVECTO®, Merck Animal Health) to achieve a minimum dose of at least 25 mg/kg. Dogs were fed one hour prior to treatment and an estimate was made of the amount of food consumed prior to dosing. Dogs in treatment group 2 served as untreated controls. Dogs were observed following treatment for any adverse events associated the treatments.

### Adulticide and egg production efficacy evaluations

On Days −2, 28, 56, 84, 91, 98, 105, 112, and 120 post-treatment, each dog was infested with approximately 200 unfed cat fleas, *C. felis felis* (KS1 strain). Then 48 h after treatment and 48 h after each subsequent infestation, dogs were removed from their individual kennels and placed into individual stainless steel metabolic cages with expanded metal floors over solid collecting pans to conduct egg collections. Dogs were housed in these cages for 3 h and were then brushed to dislodge any remaining flea eggs. After this, dogs were combed to remove any remaining fleas- as previously described- and returned to their individual kennels.

Once dogs were removed from the cages all flea eggs deposited in the stainless steel pans were collected, counted and viability of eggs determined. Eggs from each dog (up to 100) were placed in a glass Petri dish containing growth media (sand, ground dog chow, brewer’s yeast and dried blood) and held in a growth rearing chamber (Model # I30BLLC8, Percival Manufacturing Co., Boone, IA; 27-28 ° C, 70-80 % relative humidity, 24 h dark). Between 10 – 12 days after egg collection, pupae (and any larvae that had not completed cocoon formation) were sifted from the media and placed into plastic vials with lids. Adult emergence was determined by counting emerged adult fleas approximately 28 days after egg collection. Personnel conducting comb counts, egg counts, egg collections and viability assessments were blinded to treatment groups.

### Data analysis

Data at each time point were analyzed separately. The adult flea and egg count data were transformed prior to analysis using the Y = log_e_(× + 1) transformation. The log transformed data were analyzed by a mixed linear model including treatment as the fixed effect; and block as the random effect. Least squares means were used for treatment comparisons and were back transformed to obtain the estimates of geometric mean flea and egg counts. A two-tailed test was used for the comparison between groups and significance was declared when *p* < 0.05. The primary software was SAS version 9.3.

Percent control of adult fleas was calculated using geometric means with Abbott’s formula:

Efficacy (%) = 100 × (M_C_ - M_T_) / M_C_

Where: − M_C_ is the geometric mean number of total adult live fleas on untreated dogs

- M_T_ is the geometric mean number of total adult live fleas on treated dogs.

Efficacy was calculated using both geometric and arithmetic means; however, geometric means were considered as the primary approach for effectiveness evaluation.

The control of egg production was also calculated using Abbott’s formula.

## Results

On study day 0, dogs were between 7 – 7.5 months of age, with dogs in the negative control group and fluralaner treatment group weighing an average of 8.6 kg (7.4-9.7 kg) and 8.2 kg (7.5-9.0 kg), respectively. All treatment group 1 dogs were administered a 250 mg chewable tablet of fluralaner producing a mean dosage of 30.8 mg/kg (27.8-33.3 mg/kg). The fluralaner treated dogs consumed on average approximately 1 cup (0.24 L) of dry dog food in the hour prior to dosing, with two dogs only consuming a half (0.5) a cup (0.12 L) and the largest amount being 2.5 cups (0.6 L) of food by one dog.

All dogs included in the study demonstrated adequate pre-treatment flea retention with Day −4 geometric mean flea counts in treated and control groups averaging 76.0 and 78.1, respectively. Control dogs also maintained adequate infestations throughout the post-treatment period study with geometric mean flea counts ranging from 47.2 – 93.9.

The administration of a single oral dose of fluralaner produced highly significant reductions in geometric mean flea counts on treated dogs throughout the entire 122 days of the study (*P* < 0.001; Table 1). Treatment with fluralaner provided a 100 % reduction in flea counts 48 h after treatment and a 100 % reduction in flea counts within 48 h at every post-treatment infestation of the 122-day study (Table 1), an efficacy that was significant (*P* < 0.001) at every time point.

**Table 1:** Geometric mean flea counts and percent control against the KS1 cat flea strain 48 h post-treatment or infestation of dogs treated with fluralaner flavored chews

	Day 2		Day 30		Day 58		Day 86		Day 93		Day 100		Day 107		Day 114		Day 122	
Treatment^1^	Mean # of fleas^2,3^	% control^4^	Mean # of fleas	% control	Mean # of fleas	% control	Mean # of fleas	% control	Mean # of fleas	% control	Mean # of fleas	% control	Mean # of fleas	% control	Mean # of fleas	% control	Mean # of fleas	% control
Controls	93.9		75.7		78.6		47.2		55.6		62.3		54.9		54.6		52.0	
Fluralaner	0.0^a^	100	0.0^a^	100	0.0^a^	100	0.0^a^	100	0.0^a^	100	0.0^a^	100	0.0^a^	100	0.0^a^	100	0.0^a^	100

^1^12 dogs were used in this study. The 6 dogs in each control group received no treatment. The 6 dogs in the fluralaner group were administered an oral chewable tablet once on Day 0

^2^Each dog was infested with approximately 200 adult Ctenocephalides felis felis from the KS1 strain on days −2, 7, 28, 56, 84, 91, 105, 112 and 120 post-treatment

^3^Geometric mean # of live fleas recovered from dogs per treatment group

^4^ % control = ((geometric mean count control -geometric mean count treatment)/ geometric mean count treatment)) × 100

^a^geometric mean of treatment group was significantly different from control (P <0.001)

The 3-h egg collection numbers from control dogs were variable, but adequate throughout the study (Table 2). Following the Day −2 infestation when eggs were collected 48 h after treatment the geometric mean flea egg count from controls was 171.7 (Table 2). Following the post-treatment infestations geometric mean egg counts from control dogs ranged from a low of 26.7 on Day 114 to a high of 128.9 on Day 30 (Table 2).

**Table 2:** Geometric mean egg counts and percent control against the KS1 cat flea strain 48 h post-treatment or infestation of dogs treated with fluralaner flavored chews

	Day 2		Day 30		Day 58		Day 86		Day 93		Day 100		Day 107		Day 114		Day 122	
Treatment^1^	Mean # of eggs^2,3^	% control^4^	Mean # of eggs	% control	Mean # of eggs	% control	Mean # of eggs	% control	Mean # of eggs	% control	Mean # of eggs	% control	Mean # of eggs	% control	Mean # of eggs	% control	Mean # of eggs	% control
Controls	171.7		75.7		78.6		47.2		55.6		62.3		54.9		54.6		52.0	
Fluralaner	0.0^a^	100	0.0^a^	100	0.0^a^	100	0.0^a^	100	0.0^a^	100	0.0^a^	100	0.0^a^	100	0.0^a^	100	0.0^a^	100

^1^12 dogs were used in this study. The 6 dogs in each control group received no treatment. The 6 dogs in the fluralaner group were administered an oral chewable tablet once on Day 0

^2^Each dog was infested with approximately 200 adult Ctenocephalides felis felis from the KS1 strain on days −2, 7, 28, 56, 84, 91, 105, 112 and 120 post-treatment, flea eggs were collected 48 h post-treatment or post-infestation during a 3-h collection period

^3^Geometric mean # of eggs recovered from dogs per treatment group

^4^ % control = ((geometric mean count control -geometric mean count treatment)/ geometric mean count treatment)) × 100

^a^geometric mean of treatment group was significantly different from control (P ≤0.001)

Egg production from fluralaner treated dogs was reduced by 99.9 % (two eggs were collected from one dog) within 48 h after treatment. Thereafter not a single egg (100 % efficacy) was collected from any treated dog for the remainder of the 122-day study (Table 2). Egg production from the fluralaner-treated dogs was significantly lower than those for non-treated controls at all post-treatment evaluations (*P* < 0.001, Table 2).

The two eggs collected from the single fluralaner treated dog 48 h after treatment did not produce any adult fleas. Thereafter, because there were no eggs collected from any treated dog no assessment of egg viability from fluralaner treated dogs could be performed. Eggs from control dogs were viable with adult emergence always ≥46.6 % for the entire 122-day study.

There were no product related or serious adverse events observed in either the treated or control dogs.

## Discussion

An effective flea adulticide should have sufficient residual speed of flea kill to rapidly eliminate most if not all the newly emerging fleas that jump onto the treated pet between labeled administration periods. If a flea product kills those fleas before eggs are laid or has an ovicidal effect upon any eggs produced by any female flea surviving the adulticide, that product would markedly suppress the reproductive success of a flea population [7, 20].

Most currently available residual topical spot-on and systemic insecticide formulations are labelled to provide 30 days of effective flea control. Whereas, a single oral administration of fluralaner provided 100 % control of adult *C. felis felis* on dogs for at least 120 days after treatment. The efficacy duration achieved in this study was a month beyond the labeled reapplication period of 84 days and this study did not reach the end point where fleas could survive long enough to reproduce.

The first flea infestation was applied 48 h before the initial treatment. Therefore, some flea egg production is to be expected at the start of the study. However, within 48 h of fluralaner administration, egg production almost completely halted (99.9 % reduction), and those remarkably few (two) eggs were not viable. Not a single egg was laid by fleas on treated dogs for the next 4 months. Based on this longevity of residual adulticide activity veterinarians should expect that a single oral administration of a fluralaner chewable tablet would eliminate the existing flea infestation on a dog, continue to kill newly acquired fleas and that egg production should be virtually non-existent for the next 120 days.

Consumption of blood is necessary before *C. felis felis* can initiate reproduction and egg production does not begin until 24–48 h after females take their first blood meal [1, 2]. Therefore, if a residual insecticide can kill newly acquired fleas within 24 h, egg production should be markedly reduced or halted. While this study did not specifically evaluate the residual speed of kill of fluralaner, it did demonstrate that following re-infestations from Day 7 through Day 120, fluralaner killed every female flea before any eggs were produced. Proper administration of a single dose of fluralaner to dogs in a flea infested home would therefore be expected to effectively drive the flea population to extinction. All dogs and all cats in the household must be treated appropriately in order to completely shut down flea reproduction and achieve this effective control. Because fluralaner is not approved for use in cats, another effective flea control product must be chosen for cats in the household.

Egg production from untreated controls proved to be highly variable in this study, a finding that was not completely unexpected. Flea egg collection from dogs is not as straight forward as from cats, due to fecal and urine elimination by kenneled or caged dogs. Therefore, an egg collection method was used in this study involving collection for a 3-h interval based on a prior report [21] and proved successful for valid assessment in this study. The short collection interval minimizes potential fecal and urine contamination and, given the high reproductive output of *C. felis felis* should provide adequate numbers of eggs to allow for statistical comparisons of treated and untreated groups. Even then some minimal urine and fecal contamination can still occur and result in some eggs not being collected, counted or available for viability assessments.

This study was conducted using the KS1 flea strain. Several previous studies have demonstrated that this strain has reduced susceptibility or outright resistance to carbaryl, chlorpyriphos, fenthion, fipronil, imidacloprid, permethrin, pyrethrins, and spinosad [20, 22–27].

Efficacy studies evaluating various formulations of fipronil have consistently demonstrated that the 30 day residual efficacy of fipronil has been consistently less than 90 % against the KS1 flea strain [22, 23, 28]. The present study showed that the KS1 flea strain is clearly susceptible to fluralaner. Therefore, this study further verifies previous *in vitro* research demonstrating a unique fluralaner binding site that differs from fipronil, thereby renewing interest in GABA Chloride channel antagonists as effective means of flea control [29].

## Conclusions

A single oral dose of fluralaner flavored chews provided 100 % efficacy against repeated flea infestations on dogs for 4 months. Fluralaner rapidly reduced egg production of actively reproducing female fleas and then killed every single female flea before any eggs could be produced following each subsequent re-infestation for the entire 122-day evaluation period. Given the effect fluralaner had on adult fleas and egg production; it appears this treatment can interrupt flea reproduction for at least 4 months after a single treatment and should be able to eliminate an existing flea infestation on dogs and in their in-home premises. The flea reproductive breakpoint was not reached in 122 days following a single fluralaner treatment.
