# Erratum to: Efficacy of fluralaner flavored chews (Bravecto®) administered to dogs against the adult cat flea, *Ctenocephalides felis felis* and egg production

**DOI:** 10.1186/s13071-015-1014-z

**Published:** 2015-07-31

**Authors:** Michael W. Dryden, Vicki Smith, Tashina Bennett, Lisa Math, James Kallman, Kathleen Heaney, Fangshi Sun

**Affiliations:** Department of Diagnostic Medicine/Pathobiology, Kansas State University, Manhattan, 66506, KS USA; Merck Animal Health, 2 Giralda Farms, Madison, 07940, NJ USA

Unfortunately, the original version of this article [1] contained a mistake. Table 2 (table 1 here) was incorrect as it inadvertently included a duplication of data presented in Table 1. Table 2 has been corrected in the original article and is included correctly below.

**Table 1:** Geometric mean egg counts and percent control against the KS1 cat flea strain 48 h post-treatment or infestation of dogs treated with fluralaner flavored chews

	Day 2		Day 30		Day 58		Day 86		Day 93		Day 100		Day 107		Day 114		Day 122	
Treatment^1^	Mean # of eggs^2,3^	% control^4^	Mean # of eggs	% control	Mean # of eggs	% control	Mean # of eggs	% control	Mean # of eggs	% control	Mean # of eggs	% control	Mean # of eggs	% control	Mean # of eggs	% control	Mean # of eggs	% control
Controls	171.7		128.9		83.9		67.1		87.4		53.8		64.4		26.7		67.4	
Fluralaner	0.2^a^	99.9	0.0^a^	100	0.0^a^	100	0.0^a^	100	0.0^a^	100	0.0^a^	100	0.0^a^	100	0.0^a^	100	0.0^a^	100

^1^12 dogs were used in this study. The 6 dogs in each control group received no treatment. The 6 dogs in the fluralaner group were administered an oral chewable tablet once on Day 0

^2^Each dog was infested with approximately 200 adult Ctenocephalides felis felis from the KS1 strain on days—2, 7, 28, 56, 84, 91, 105, 112 and 120 post-treatment, flea eggs were collected 48 h post-treatment or post-infestation during a 3-h collection period

^3^Geometric mean # of eggs recovered from dogs per treatment group

^4^% control = ((geometric mean count control -geometric mean count treatment)/ geometric mean count treatment)) x 100

^a^geometric mean of treatment group was significantly different from control (P ≤0.001)
